# Honokiol Inhibits SARS-CoV-2 Replication in Cell Culture at a Post-Entry Step

**DOI:** 10.1128/spectrum.03273-22

**Published:** 2023-05-04

**Authors:** Clarisse Salgado-Benvindo, Anouk A. Leijs, Melissa Thaler, Ali Tas, Jack L. Arbiser, Eric J. Snijder, Martijn J. van Hemert

**Affiliations:** a Department of Medical Microbiology, Leiden University Medical Center, Leiden, The Netherlands; b Department of Dermatology, Emory University School of Medicine, Atlanta, Georgia, USA; c Division of Dermatology, Veterans Affairs Medical Center, Decatur, Georgia, USA; Cornell University College of Veterinary Medicine

**Keywords:** MERS-CoV, SARS-CoV, SARS-CoV-2, antiviral agents, coronaviruses, honokiol, inhibitor

## Abstract

Severe acute respiratory syndrome coronavirus 2 (SARS-CoV-2) emerged in 2019, and the resulting pandemic has already caused the death of over 6 million people. There are currently few antivirals approved for treatment of the 2019 coronavirus disease (COVID-19), and more options would be beneficial, not only now but also to increase our preparedness for future coronavirus outbreaks. Honokiol is a small molecule from magnolia trees for which several biological effects have been reported, including anticancer and anti-inflammatory activities. Honokiol has also been shown to inhibit several viruses in cell culture. In this study, we determined that honokiol protected Vero E6 cells from SARS-CoV-2-mediated cytopathic effect, with a 50% effective concentration of 7.8 μM. In viral load reduction assays, honokiol decreased viral RNA copies as well as viral infectious progeny titers. The compound also inhibited SARS-CoV-2 replication in the more relevant human A549 cells expressing angiotensin converting enzyme 2 and transmembrane protease serine 2. Time-of-addition and other assays showed that honokiol inhibited virus replication at a post-entry step of the replication cycle. Honokiol was also effective against more recent variants of SARS-CoV-2, including Omicron, and it inhibited other human coronaviruses as well. Our study suggests that honokiol is an interesting molecule to be evaluated further in animal studies and, when successful, maybe even in clinical trials to investigate its effect on virus replication and pathogenic (inflammatory) host responses.

**IMPORTANCE** Honokiol is a compound that shows both anti-inflammatory and antiviral effects, and therefore its effect on SARS-CoV-2 infection was assessed. This small molecule inhibited SARS-CoV-2 replication in various cell-based infection systems, with up to an ~1,000-fold reduction in virus titer. In contrast to earlier reports, our study clearly showed that honokiol acts on a postentry step of the replication cycle. Honokiol also inhibited different recent SARS-CoV-2 variants and other human coronaviruses (Middle East respiratory syndrome CoV and SARS-CoV), demonstrating its broad spectrum of antiviral activity. The anticoronavirus effect, combined with its anti-inflammatory properties, make honokiol an interesting compound to be further explored in animal coronavirus infection models.

## INTRODUCTION

Since it emerged in 2019, the severe acute respiratory syndrome coronavirus 2 (SARS-CoV-2) lead to a pandemic that involved over 500 million infections and over 6 million deaths globally. SARS-CoV-2 is a member of the *Betacoronavirus* genus within the *Coronaviridae* family, and it is genetically close to SARS-CoV, with almost 80% identity (1). Coronavirus disease of 2019 (COVID-19), the disease caused by SARS-CoV-2, often involves mild symptoms, like fever, cough, tiredness, and loss of taste or smell, but it can lead to more serious outcomes, with patients developing shortness of breath, severe pneumonia, respiratory failure, or death (2). Since it emerged in 2019, SARS-CoV-2 has given rise to innumerous variants of interest and/or concern, some of which have displayed a worryingly fast spread, increased immune or vaccine escape, and/or changes in disease severity (3–5). Risk factors for severe disease include old age, obesity, and defects in interferon signaling. Host factors are involved in pathogenesis, e.g., via the inflammatory response, but they also play a role in viral replication and therefore constitute interesting therapeutic targets.

Even with the advances in vaccine development, antivirals are still of major importance in order to treat patients that, for a variety of reasons, could not be vaccinated or that did not properly respond to vaccination. Moreover, waning immunity and the continuing emergence of new variants that, to various degrees, can escape natural or vaccine-induced immunity in the population, or the (zoonotic) emergence of yet another new coronavirus, make it even more important to increase our preparedness by developing antivirals. Preferably such antivirals should not only be active against SARS-CoV-2 but also to a broad spectrum of coronaviruses. Compared to vaccines, antivirals have advantages in terms of storage, distribution, administration, and acceptance by part of society. Currently, there are very few options available for treating COVID-19 patients, such as remdesivir, paxlovid, and molnupiravir. Issues with costs, route of administration, concerns about side effects, and possible development of resistance regarding the currently approved antivirals against SARS-CoV-2 make it necessary to continue research on potential new antivirals. Early in the pandemic, as part of our drug repurposing efforts, we identified the small molecule honokiol (HK) as an inhibitor of SARS-CoV-2 replication in cell culture. HK is a polyphenolic lignan compound, extracted from the barks of plants of the *Magnolia* genus. It has been used in traditional Chinese medicine for its analgesic and other effects (6). In Western medicine, HK has also been the subject of pharmaceutical and biomedical studies into its anticancer (7–9), anti-inflammatory (10, 11), antithrombotic (12), antioxidative (13, 14), and antiviral activities (15–17). HK has been found to modulate several molecular targets, including NF-κB, STAT3, m-TOR, and SIRT3 (8, 18–20). In this study, we focused on the efficacy of honokiol in inhibiting SARS-CoV-2 replication in cell culture. Future studies will assess its effect on the host response to the virus, which could play a role in controlling infection and mediating the severity of disease. We found that HK decreased replication of the early pandemic and more recent variants of SARS-CoV-2, as well as other pathogenic coronaviruses.

Our study provides a basis to further explore the effect of HK and analogs in *in vivo* (animal) studies to assess its effects on coronavirus infection and resulting (pathogenic) host responses.

## RESULTS

### Honokiol inhibits SARS-CoV-2 replication in Vero E6 cells.

**FIG 1:**
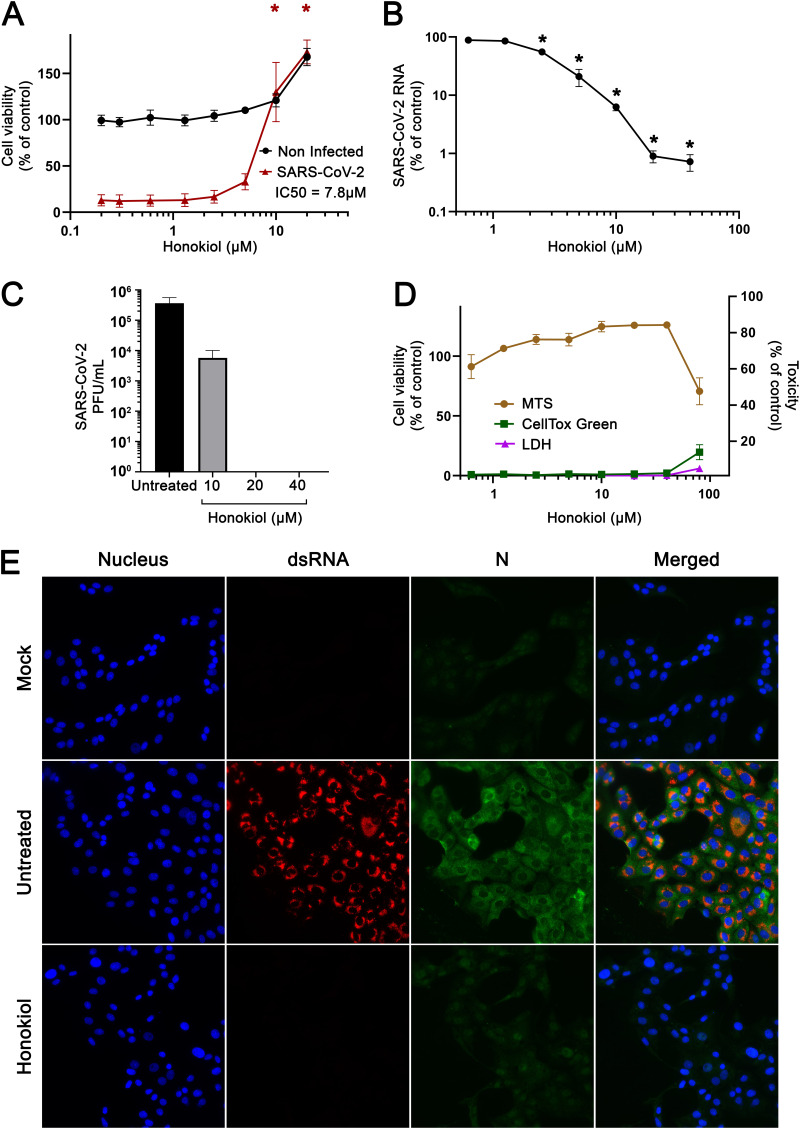
Effects of honokiol on SARS-CoV-2-mediated cytopathic effect and viral replication in Vero E6 cells. (A) Vero E6 cells were treated with increasing concentrations of HK and then infected with SARS-CoV-2 at an MOI of 0.015. After 3 days, cell viability was measured by MTS assay. The viability of noninfected cells treated with compound was determined in parallel to assess cytotoxicity of the compound. Data points represent the means ± SEM from 3 independent experiments, and analysis was done with a two-way ANOVA test, followed by Bonferroni’s *post hoc* test. (B and C) Vero E6 cells were treated with increasing concentrations of HK and, after 6 h, were infected with SARS-CoV-2 at an MOI of 1. Supernatant samples were harvested at 16 hpi to quantify SARS-CoV-2 RNA levels by RT-qPCR (B) and infectious progeny titers by plaque assay (C). The means ± SEM from 4 independent experiments are shown. Data were analyzed with one-way ANOVA, followed by Bonferroni’s *post hoc* test. (D) Vero E6 cells were treated with increasing concentrations of HK and different assays were applied to assess toxicity of the compound. Data points represent the means ± SEM from 2 independent experiments. (E) Vero E6 cells were infected with SARS-CoV-2 at an MOI of 1 in the presence or absence of 20 μM HK. After infection, treated cells were incubated for 16 h in medium with or without HK. After fixation with 3% PFA, cells were analyzed by immunofluorescence microscopy with antibodies against dsRNA and nucleocapsid protein and Hoechst to stain nuclei. Uninfected cells (mock) were included as controls.

To evaluate if HK could protect cells from SARS-CoV-2 infection, we performed a cytopathic effect (CPE) reduction assay. Treatment with HK protected infected Vero E6 cells from CPE in a dose-dependent manner, with a 50% effective concentration (EC_50_) of around 7.8 μM (Fig. 1A). In parallel in the same plate, uninfected cells were treated with the compound to assess its toxicity. We observed no signs of cytotoxicity in Vero E6 cells for the concentrations tested.

To confirm that the observed protection in the CPE reduction assays was indeed due to an inhibitory effect of HK on virus replication, we conducted viral load reduction assays. Vero E6 cells were pretreated with increasing concentrations of HK starting 6 h before they were infected with SARS-CoV-2 at a multiplicity of infection (MOI) of 1 for 1 h in the presence of the compound. After incubation with medium containing compound for 16 h, supernatant was harvested for virus quantification. Reverse transcription-quantitative PCR (RT-qPCR) analysis showed that HK caused a dose-dependent decrease in viral RNA levels. At 20 μM, a 99% reduction in viral RNA copies (from 1.6 × 10^9^ to 1.3 × 10^7^ copies/mL) was observed (Fig. 1B). Determination of the infectious virus titer in the supernatant by plaque assay showed that treatment with 20 μM HK caused a >3-log reduction in infectious virus titer, to below the limit of detection (Fig. 1C). Cell viability, measured in a 3-(4,5-dimethylthiazol-2-yl)-5-(3-carboxymethoxyphenyl)-2-(4-sulfophenyl)-2H-tetrazolium (MTS) assay, was not affected at these concentrations, and only higher concentrations showed cytotoxicity in Vero E6 cells, with a 50% cytotoxic concentration (CC_50_) of ≈34 μM. Toxicity at concentrations higher than 40 μM HK was also observed, although to a lesser extent, when other toxicity assays (CellTox green and lactate dehydrogensae [LDH] assays) were performed (Fig. 1D). Since the MTS assay had the best sensitivity and to avoid underestimation of toxicity, the MTS assay was used throughout this study in order to assess HK’s cytotoxicity.

The antiviral effect of HK was also assessed by immunofluorescence assay on Vero E6 cells infected with SARS-CoV-2 at an MOI of 1 in the presence or absence of 20 μM HK (Fig. 1E). Samples were fixed at 16 h postinfection (hpi), and staining for double-stranded RNA (dsRNA) and viral N protein showed widespread infection in untreated cultures with almost all cells positive. In the HK-treated samples, we could not detect any dsRNA and very little N signal, similar to the signals in uninfected cells. This provided additional evidence for the antiviral effect of HK.

Together, all these data suggest that HK inhibits SARS-CoV-2 replication and protects cells from virus-induced CPE, without causing measurable cytotoxicity at concentrations up to 20 μM.

### Honokiol inhibits SARS-CoV-2 replication at a post-entry step of the replication cycle.

**FIG 2:**
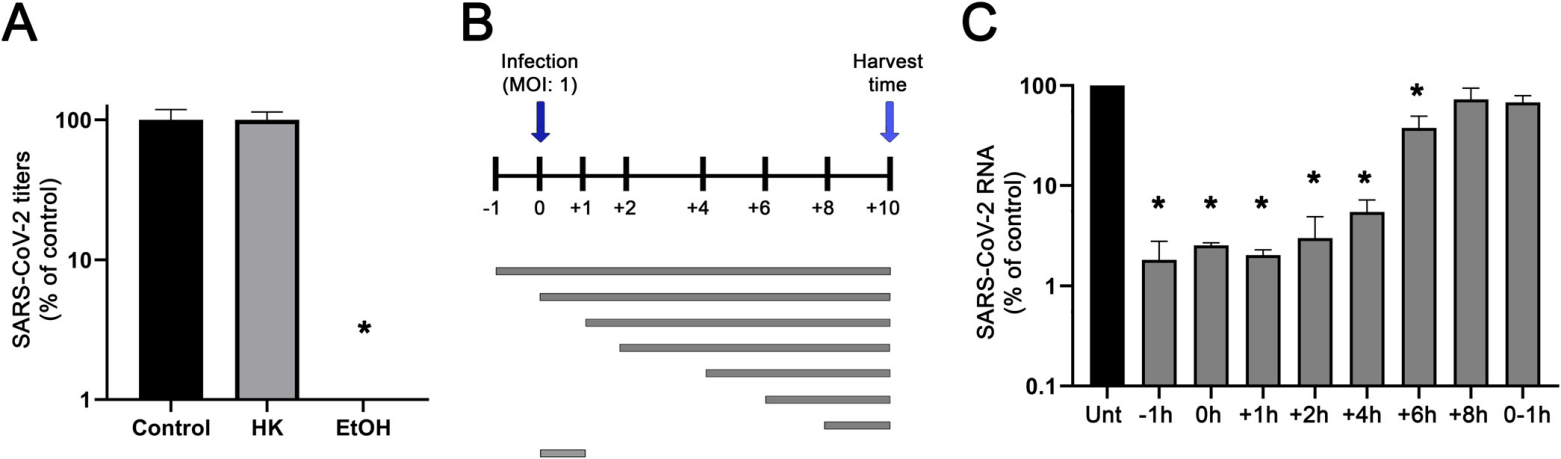
HK inhibits SARS-CoV-2 replication at a postentry step of the replication cycle. (A) Assessment of the virucidal potential of HK. A SARS-CoV-2 stock was incubated in medium (control) with 50 μM HK or 70% ethanol. The remaining infectious virus titers were determined by plaque assay and normalized to that of the untreated control (100%). The means ± SEM from 3 independent experiments are shown, and data were analyzed by one-way ANOVA and Bonferroni’s *post hoc* test. (B) Schematic representation of the time-of-addition assay, depicting the different treatment intervals during which infected Vero E6 cells were exposed to 20 μM HK. (C) At 10 hpi, supernatants were harvested and viral load was determined by quantifying extracellular viral RNA copies by RT-qPCR. Copy numbers were normalized to the level of untreated infected cells (100%). The means ± SEM from 3 independent experiments are shown, and data were analyzed by one-way ANOVA and Bonferroni’s *post hoc* test.

To understand which step of the replication cycle is inhibited by HK, we first assessed whether the compound has virucidal properties. We therefore incubated a virus stock for 1 h with 50 μM HK or 70% ethanol (Fig. 2A). As expected, the control treatment with 70% ethanol reduced the infectious virus level to below the limit of detection (>4-log reduction). After a 1-h treatment with a high dose of HK, the infectious virus titer remained at the level of untreated virus stocks. This demonstrated that HK had no virucidal properties, so is not acting directly on the virus particle. To pinpoint which step of the viral replication cycle is inhibited by HK, a time-of-addition assay was performed. Treatment of Vero E6 cells with 20 μM HK was initiated at different time points, after which it remained present until the end of the assay unless indicated otherwise (Fig. 2B). At 0 h, cells were infected with SARS-CoV-2 at an MOI of 1, and at 10 hpi supernatant was harvested for virus quantification. Initially, we assessed the effects of different pretreatments with 20 μM HK, and we did not observe any difference in effectiveness between treatments initiated at any time point between 8 and 1 h prior to infection (data not shown). We then performed assays with treatments initiated at 1 h before infection or at 0, 1, 2, 4, 6, or 8 hpi, followed by quantification of extracellular viral RNA levels at 10 hpi by RT-qPCR. The maximum effect of the compound was still observed when treatment was initiated as late as 4 h postinfection. When treatments were started later, HK gradually lost its inhibitory effect and was no longer effective after 8 hpi (Fig. 2C). When the compound was only present from 0 to 1 hpi, it had no inhibitory effect, suggesting it does not interfere with the early steps of the replication cycle.

**FIG 3:**
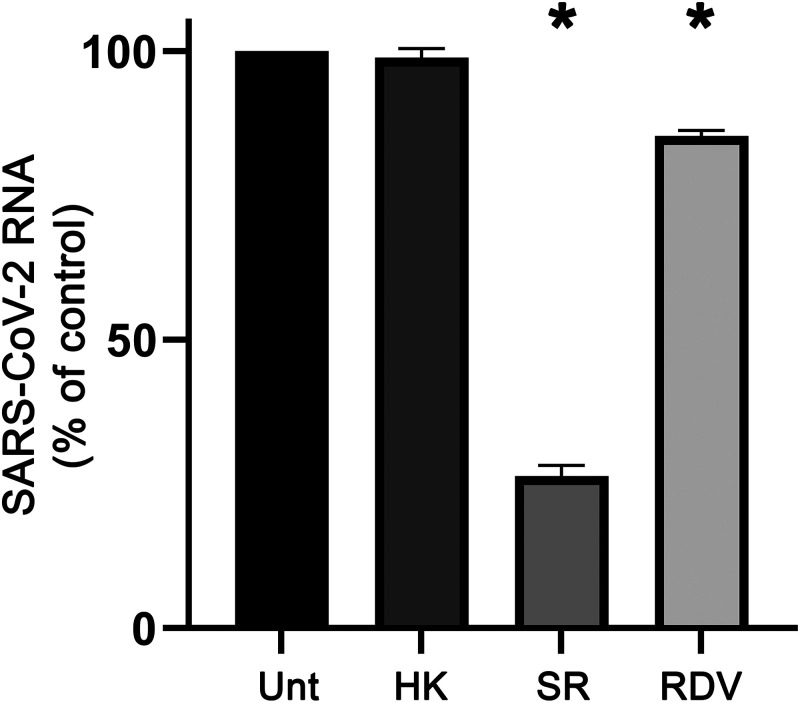
Effect of HK on the early steps of the SARS-CoV-2 replication cycle. Vero E6 cells were infected at an MOI of 5 and were either left untreated (Unt) or were treated with 20 μM honokiol (HK), 100 μM suramin (SR), or 25 μM remdesivir (RDV). At 2 hpi, intracellular viral RNA levels were quantified by internally controlled multiplex RT-qPCR. Copy numbers were normalized to the levels in untreated infected cells (100%). The means ± SEM from 3 independent experiments are shown, and data were analyzed by one-way ANOVA and Bonferroni’s *post hoc* test.

Our time-of-addition analysis showed that HK had no effect on the early steps of infection, in contrast to what was claimed in another study. Therefore, we performed an additional experiment to validate our findings. We focused on the early events in the replication cycle by quantifying the amount of intracellular viral RNA at a very early time point, i.e., 2 h postinfection, of Vero E6 cells infected at an MOI of 5 for 1 h. Cells were left untreated or were treated with 20 μM HK, 100 μM of the entry inhibitor suramin as positive control (21), or 25 μM of the nucleoside analog remdesivir as a negative control (22). Cells treated with suramin during infection accumulated 80% less intracellular viral RNA at 2 hpi than untreated cells (Fig. 3). At 2 hpi, cells treated with the polymerase inhibitor remdesivir displayed only slightly lower intracellular RNA levels compared to untreated cells, in line with its mechanism of action, which does not involve binding or entry. The slightly lower RNA levels might have been due to effects on early RNA synthesis.

Treatment of cells with HK during the first 2 h of infection had no effect on the amount of viral RNA that accumulated in the cells at 2 hpi compared to that in untreated cells, demonstrating that HK had no effect on binding or entry.

### Honokiol inhibits SARS-CoV-2 replication in human lung cells.

**FIG 4:**
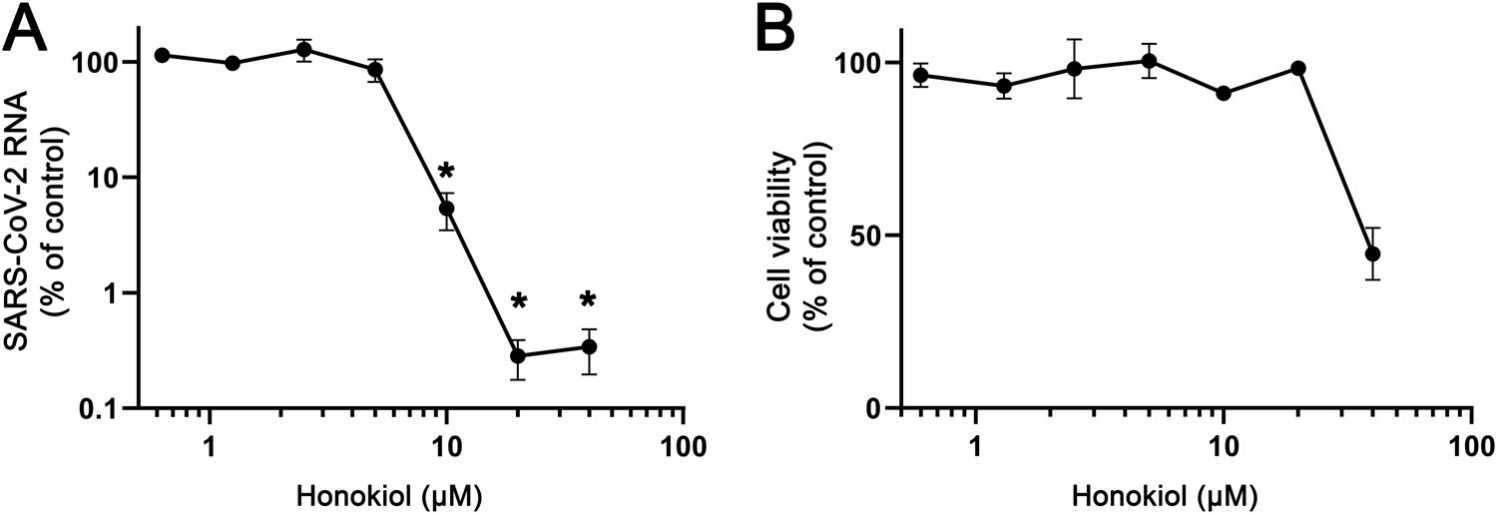
Effect of HK on SARS-CoV-2 replication in A549-ACE2-TMPRSS2 cells. (A) A549 cells expressing ACE2 and TMPRSS2 were infected with SARS-CoV-2 at an MOI of 1. Treatment with HK was initiated 6 h prior to infection, and the compound remained present until medium was harvested at 16 hpi to quantify extracellular viral RNA levels by RT-qPCR. Copy numbers were normalized to the level in untreated infected cells (100%). The means ± SEM from 3 independent experiments are shown, and data were analyzed by one-way ANOVA and Bonferroni’s *post hoc* test. (B) Compound toxicity was assessed in parallel by MTS assay on uninfected cells treated with HK.

To evaluate if HK can also inhibit SARS-CoV-2 in a more relevant cell model, human A549-ACE2-TMPRSS2 cells were used. In a viral load reduction assay, similar to the one described for Vero E6 cells, A549-ACE2-TMPRSS2 cells were pretreated with HK at increasing concentrations and subsequently infected at an MOI of 1. RT-qPCR analysis of supernatant samples harvested at 16 hpi showed that SARS-CoV-2 was also inhibited in this infection model. Treatment with 10 μM HK led to a 2-log decrease in copy numbers (Fig. 4A). Measurement of cell viability showed that, as for Vero E6 cells, 20 μM could be considered a noncytotoxic concentration, whereas at 40 μM toxicity clearly became an issue with this cell line (Fig. 4B). These results suggested that the effect of HK is cell line independent and can be reproduced in different infection models.

### Honokiol is effective against different SARS-CoV-2 variants of concern.

**FIG 5:**
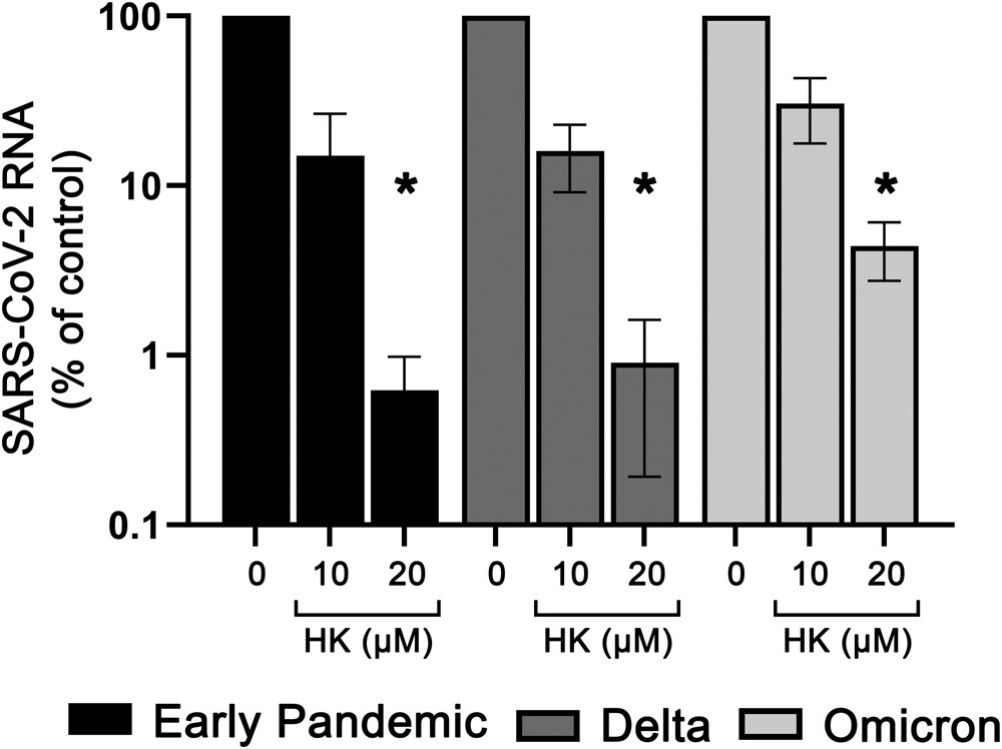
Effect of HK on SARS-CoV-2 variants of concern. Vero E6 cells were treated with 10 or 20 μM HK for 6 h and subsequently infected with a SARS-CoV-2 variant at an MOI of 1, followed by incubation for 16 h in the presence of the compound. At 16 hpi, supernatant was harvested and RT-qPCR was used to quantify the extracellular viral RNA levels. Copy numbers were normalized to the level of untreated infected cells (100%). The means ± SEM from 3 independent experiments are shown, and data were analyzed by two-way ANOVA and Bonferroni’s *post hoc* test.

To investigate if HK was also able to inhibit replication of SARS-CoV-2 variants other than the original early pandemic strain that was used throughout this study, we tested its efficacy against the two major variants of concern circulating at the time of this project, namely, Delta (B.1.617.2) and Omicron (B.1.1.529). Vero E6 cells were treated with HK for 6 h and were infected with each variant at an MOI of 1. At 16 hpi, medium was harvested for determination of viral load by RT-qPCR (Fig. 5). At 20 μM, HK inhibited all three variants, by at least 95%.

### Honokiol inhibits replication of a broad spectrum of coronaviruses.

**FIG 6:**
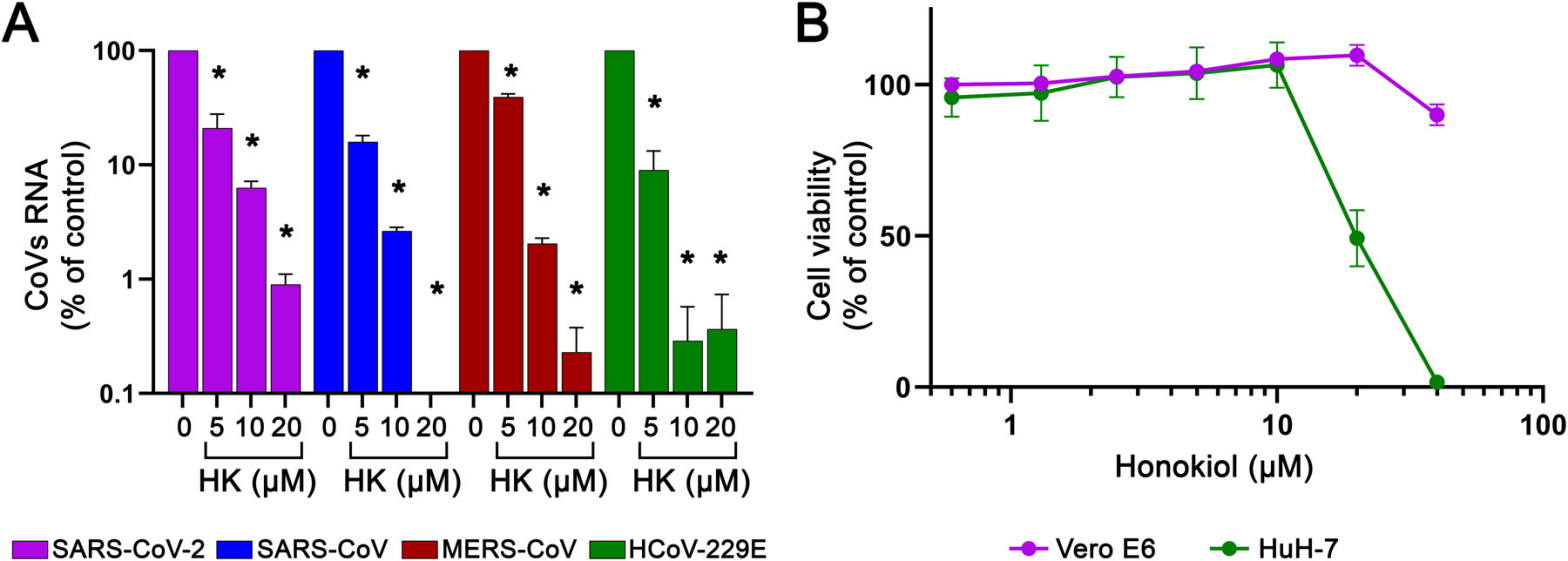
Effect of HK on various human coronaviruses. (A) Vero E6 cells were infected with SARS-CoV or SARS-CoV-2, and HuH-7 cells were infected with MERS-CoV or HCoV-229E at an MOI of 1. HK was added 6 h before infection and remained present until the time of harvest at 16 hpi for SARS-CoV, SARS-CoV-2, and MERS or at 24 hpi for HCoV-229E. Medium was collected and the levels of viral RNA were quantified by RT-qPCR. Copy numbers were normalized to the level in untreated infected cells (100%). The means ± SEM from 3 independent experiments are shown, as analyzed by two-way ANOVA and Bonferroni’s *post hoc* test. (B) A viability assay (MTS) was done in parallel to determine the compound’s cytotoxicity in Vero E6 and HuH-7 cells. The means ± SEM from 6 independent experiments are shown.

To evaluate HK’s effectiveness against other relevant human coronaviruses, we used Vero E6 cells infected with SARS-CoV or SARS-CoV-2 and HuH-7 cells infected with Middle East respiratory syndrome coronavirus (MERS-CoV) or human coronavirus type 229E (HCoV-229E). Infections were done at an MOI of 1 following a 6-h pretreatment with HK, after which the compound remained present until the end of the experiment. Medium from MERS-CoV-, SARS-CoV-, and SARS-CoV-2-infected cells was harvested for virus quantification at 16 hpi, and medium from HCoV-229E-infected cells was harvested at 24 hpi. RT-qPCR analysis showed that all viruses were inhibited by HK in a dose-dependent manner (Fig. 6A), with EC_90_ values of 4.5, 5.8, and 5.7 μM for HCoV-229E, MERS-CoV, and SARS-CoV, respectively. It is important to note that the maximum nontoxic dose of HK varied depending on the cell line used, with HK showing a decrease in viability of around 50% in HuH-7 cells already at 20 μM (CC_50_ of 20 μM) (Fig. 6B), while this concentration was not cytotoxic in Vero E6 or A549-ACE2-TMPRSS2 cells. However, the antiviral effect of HK was observed at lower doses in HuH-7 cells than in the other cells, as HuH-7 cells infected with MERS-CoV or HCOV-229E displayed a >95% reduction in viral RNA copies when cells were treated with 10 μM of the compound. These data indicated that, despite some differences in toxicity, HK inhibited coronavirus replication in a cell line-independent manner and displayed a broad-spectrum antiviral effect against a range of different pathogenic human coronaviruses, i.e., SARS-CoV-2, MERS-CoV, SARS-CoV, and HCoV-229E.

## DISCUSSION

Honokiol is a small lignan compound that is extracted from the barks, cones, and leaves of trees of the *Magnolia* genus. These plants have been used in traditional Chinese medicine to relieve anxiety, depression, and pain. In Western medicine, anti-inflammatory, antithrombotic, antioxidative, antifungal, antiarrhythmic, and, mainly, antitumor properties have been attributed to HK (23). HK is thought to inhibit tumor progression through modulation of different signaling pathways that behave aberrantly in cancer patients. For example, HK can induce autophagy in different cancer cells by downregulating the PI3K/Akt/mTOR signaling pathway (8, 24). HK is also able to downregulate NF-κB and STAT3 (18, 20), both of which are generally involved in tumor promotion (25). Other targets of HK include Sirt3 (19), Nrf2 (26), MAPK (27), and SMAD (28) signaling pathways. Some of these factors, such as Nrf2, Sirt3, and mTOR (29–31), have been linked to pathways involved in antiviral responses. HK has also been shown to possess antiviral effects against a broad range of different viruses, such as hepatitis C virus (16), dengue virus (15), herpes simplex virus 1 (17), Usutu virus (32), and Mayaro virus (33). This provided the rationale for assessing the effect of HK on SARS-CoV-2 infection in cell culture. We demonstrated that in CPE reduction assays HK protected cells from the virus-mediated CPE in a dose-dependent manner (Fig. 1), with an EC_50_ of 7.8 μM. The effect of HK was cell line independent, as it inhibited SARS-CoV-2 replication in both African green monkey Vero E6 and human A549 cells expressing ACE2 and TMPRSS2 (Fig. 2 and 4).

HK has no virucidal effects, and our (single-cycle) time-of-addition analysis revealed that HK retained its full inhibitory effect even when treatment was initiated as late as 4 hpi and then gradually lost effectiveness when treatment was initiated later. When the compound was only present during the 1 h of infection, it had no antiviral effect. To validate that HK had no effect on binding or entry, we focused on the analysis of these early steps by measuring the amount of viral RNA that was present in cells very early in infection, at 2 hpi. Suramin, a known inhibitor of SARS-CoV-2 entry (21), caused an 80% reduction in early viral RNA levels compared to untreated cells. Treatment of cells with HK caused no reduction in early intracellular viral RNA levels. Together, our results suggest that HK inhibits a postentry step of the replication cycle. In contrast to our observations, two previous studies suggested that HK or its analogs can inhibit SARS-CoV-2 infection by targeting the binding and entry steps of the replication cycle. One study suggested that Spike-ACE2 binding was inhibited (34). However, this study was not performed in SARS-CoV-2-infected cells and instead used an artificial system with pseudotyped viruses and biochemical assays. In these other studies, less than 50% inhibition was observed at a high dose (50 μM) of HK. Another study (35) suggested that HK inhibits furin-like proteases, but the specificity and efficacy of this inhibition (~30% at 100 μM) remain debatable, considering that a known furin inhibitor was ~700 times more potent in the same study.

We assessed HK’s spectrum of activity and observed that it also inhibited MERS-CoV, SARS-CoV, and HCoV-229E replication in cell culture, in line with the idea that HK likely exerts its antiviral effect through one or more host factors. The fact that HK also inhibited MERS-CoV and HCoV-229E, which use DPP4 and human aminopeptidase N as receptors, respectively (36, 37), also suggests that it is unlikely that targeting of ACE2 by HK is responsible for the observed antiviral effect. Previous studies have shown that HK can increase the expression of interferon and interferon-stimulated genes, like ISG15, OAS2, and inflammatory cytokines like tumor necrosis factor alpha and intereukin-1β (33, 38). Therefore, we hypothesize that HK inhibits coronavirus replication via host factors involved in one or more of the pathways involved in inflammation and/or innate immune responses. Since HK is active in Vero E6 cells, which are defective in interferon signaling, we hypothesize that an interferon-independent mechanism is involved.

The massive scale of the SARS-CoV-2 pandemic, complications with (global) vaccine roll out, and the continuing emergence of variants (of concern) that can escape natural or vaccine-induced immunity all stress the importance of developing (multiple) antivirals to increase our preparedness. Direct-acting antivirals are a good option, but their spectrum of activity and development of resistance are concerns. Therefore, also compounds that modulate pathways that are involved in the replication of (a broad range of) viruses are interesting candidates to explore as potential antivirals, in particular when this involves (repurposing of) existing compounds with favorable pharmacokinetics and safety profiles. HK inhibited various pathogenic coronaviruses, including the two major SARS-CoV-2 variants that were circulating at the time of our study, i.e., the Delta and Omicron variants (Fig. 5).

HK appears to be well-tolerated in animal models, has a half-life of almost 5 h (39), and in mice free HK levels in the plasma reached around 200 μg/mL (40). This would be well above the EC_50_ that we found in our study (around 5 μg/mL) and suggests that efficacy studies in animal models could be feasible, with the side note that EC_50_ values from cell-based assays cannot be simply extrapolated to efficacy in animal models. Some clinical studies have been conducted in humans subjected to HK or whole magnolia bark extract treatments (reviewed in reference 41). In one study, three volunteers of the treatment group dropped out due to side effects, while the other 16 subjects completed the study without any signs of serious adverse events (42). Although these studies suggested that HK can be used in humans, we are absolutely not advocating the use of honokiol as treatment for coronavirus infections, based on the results of our study. The compound should first be tested for safety and efficacy in animal models and, in case of positive results, in properly conducted clinical trials. Effective treatment of SARS-CoV-2 infection will require efficient antiviral agents, as well as agents that modify the host response. Human aging is associated with a more severe inflammatory response to SARS-CoV-2, and Sirt3 activators such as honokiol have anti-inflammatory effects *in vivo*, which could have the additional benefit of reducing pathological inflammation. In conclusion, HK’s broad-spectrum antiviral effect against multiple coronaviruses and possible effects on inflammation and the innate immune response (as seen by others) make it an interesting compound to explore in animal studies and maybe ultimately in clinical trials as (part of) treatment for coronavirus infections.

## MATERIALS AND METHODS

### Cell culture, compounds, and viruses.

Vero E6 cells were maintained in Dulbecco’s modified Eagle’s medium (DMEM; Lonza), supplemented with 8% fetal calf serum (FCS; Bodinco), 2 mM l-glutamine, 100 IU/mL of penicillin, and 100 μg/mL of streptomycin (Sigma-Aldrich). A549 cells expressing ACE2 and TMPRSS2 (here referred to as A549-ACE2-TMPRSS2 cells) were a kind gift from Stuart Neil (King's College, London, United Kingdom) and are described elsewhere (43). These cells were maintained in DMEM supplemented with 8% FCS, 100 IU/mL of penicillin, 400 μg/mL of G418 (InvivoGen), and 1 μg/mL of puromycin (Sigma-Aldrich). Huh-7 cells were grow in DMEM supplemented with 8% FCS, 2 mM l-glutamine, nonessential amino acids, 100 IU/mL of penicillin, and 100 μg/mL of streptomycin.

The SARS-CoV-2/Leiden-0002 (GenBank accession number MT510999.1) and SARS-CoV-2/Leiden-0008 (GenBank accession number MT705206.1) isolates were obtained from nasopharyngeal samples at the Leiden University Medical Center (LUMC) during the first wave of the pandemic. For infections of A549-ACE2-TMPRSS2 cells, a SARS-CoV-2 isolate that was adapted to this cell line was used (unpublished data). The SARS-CoV-2 Delta variant (Leiden-KUL-Delta1) and Omicron variant (Leiden-O-71084/2021) were isolated at LUMC from a local clinical sample and material kindly provided by the National Institute for Public Health and the Environment (RIVM, Netherlands), respectively. MERS-CoV Jordan-N3 (GenBank accession number KJ614529.1), SARS-CoV/Frankfurt-1 (GenBank accession number AY291315.1), and HCoV-229E (GenBank accession number NC_002645.1) were also used for this study. Infections were done using Eagle’s minimal essential medium with 25 mM HEPES (Lonza) supplemented with 2% FCS, 2 mM l-glutamine, 100 IU/mL of penicillin, and 100 μg/mL of streptomycin. All experiments with SARS-CoV, SARS-CoV-2, and MERS-CoV were done in the LUMC biosafety level 3 facilities, while HCoV-229E infections were done in a biosafety level 2 laboratory.

Honokiol was purchased from MedChem Express as a powder and dissolved in dimethyl sulfoxide.

### CPE reduction assay.

Vero E6 cells were seeded in 96-well clusters at a density of 5 × 10^3^ cells/well in 100 μL. Twenty-four hours after seeding, cells were incubated with 2-fold dilutions of compound for 1 h. After that, half the cells were left uninfected, for analysis of the compound’s toxicity, or infected with SARS-CoV-2 at a low MOI of 0.015. After 3 days, cell viability was measured by MTS assay using the CellTiter 96 AQueous MTS reagent (Promega). MTS absorbance was measured at 495 nm with an EnVision multiplate reader (PerkinElmer). A CellTox Green cytotoxicity assay (Promega) and CyQuant LDH cytotoxicity assay (Thermo Fisher Scientific) were used to evaluate the compound’s toxicity.

### Viral load reduction assay.

Cells were seeded at a density of 1 × 10^4^ (Vero E6 and HuH-7) or 2 × 10^4^ (A549-ACE2-TMPRSS2) cells per well in 100 μL medium in a 96-well cluster. Twenty-four hours after seeding, cells were treated with increasing concentrations of the compound and were incubated for 6 h at 37°C. Subsequently, cells were infected for 1 h with virus at an MOI of 1. Supernatant was harvested at 16 hpi for SARS-CoV, SARS-CoV-2, and MERS-CoV infection or at 24 hpi for HCoV-229E experiments. To assess viral load, extracellular viral RNA copies were quantified by RT-qPCR, and/or infectious progeny was quantified by plaque assay. The potential cytotoxicity of the compound was always tested in parallel by MTS assay in equally treated, but uninfected, cells.

### Plaque assay.

Vero E6 cells were seeded 1 day before infection in regular culture medium at 1.5 × 10^4^ cells/well in 1 mL in 12-well clusters. On the day of the assay, 10-fold serial dilutions of samples were prepared in infection medium. These dilutions were used as inoculum to infect cells for 1 h at 37°C, after which inoculum was removed and replaced with overlay medium containing 1.2% Avicel, 1% antibiotics, 2% FCS, and 50 mM HEPES in DMEM. Cells were incubated at 37°C for 3 days, and clusters were fixed with 7.4% formaldehyde. Wells were stained with crystal violet, and plaques were manually counted to determine the sample’s infectious virus titer.

### RNA isolation and RT-qPCR.

RNA was isolated from cell culture supernatants using the Bio-on-Magnetic-Beads (BOMB) method (44) using a Viaflo Assist Plus robotic system (Integra), following sample lysis in a buffer containing 3 M guanidine-thiocyanate, 2% *N*-lauroyl-sarcosine sodium salt, 1 M Tris-HCl (pH 7.6), and 0.5 M EDTA. Equine arteritis virus RNA was spiked into the lysis reagent as an internal technical control for RNA isolation efficiency and quality. Viral RNA was amplified by RT-qPCR using the TaqMan Fast Virus 1-step master mix (Thermo Fisher Scientific). Primers and probes targeting SARS-CoV-2 RNA-dependent RNA polymerase have been described elsewhere (21) and were also used for SARS-CoV quantification. MERS-CoV and HCoV-229E primer sets were designed in-house, targeting the gene for the nucleoprotein (N) of each virus. For absolute quantification, a standard curve generated from a T7 RNA polymerase *in vitro* transcript containing the necessary RT-qPCR target fragments was used. The reaction was performed in a CFX384 Touch real-time PCR detection system (Bio-Rad, Netherlands), with a program of 5 min at 50°C and 20 s at 95°C, followed by 45 cycles of 5 s at 95°C and 30 s at 60°C.

### Immunofluorescence assay.

Vero E6 cells grown on glass coverslips were infected with SARS-CoV-2 at an MOI of 1, in the presence or absence of 20 μM HK. After 16 h, cells were fixed with 3% paraformaldehyde (PFA) in phosphate-buffered saline (PBS). Coverslips were then rinsed 3× with PBS and permeabilized for 10 min with 0.2% Triton X-100 in PBS. Cells were incubated with primary antibodies in PBS with 5% FCS for 1 h at room temperature. Primary antibodies used were a mouse monoclonal anti-dsRNA J2 (45) and a rabbit anti-SARS-CoV nucleocapsid (46). Coverslips were washed and incubated with secondary antibodies: an Alexa488-conjugated goat anti-rabbit IgG antibody (ThermoFisher) and a Cy3-conjugated donkey anti-mouse IgG antibody (Jackson ImmunoResearch Laboratories). Nuclei were stained with Hoechst 33258 (ThermoFisher). After mounting with Prolong Gold (ThermoFisher), coverslips were imaged using a Leica DM6B fluorescence microscope and LASX software.

### Virucidal activity determination.

A total of 1 × 10^6^ PFU of SARS-CoV-2 was incubated with infection medium (control), HK (to a final concentration of 50 μM), or ethanol (70% final concentration) for 1 h at 37°C. Samples were then serially diluted 10-fold in infection medium, and the (remaining) infectious virus titer was determined by plaque assay. Only samples that were diluted 100-fold or more were analyzed by plaque assay to avoid unintended antiviral or cytotoxic effects arising from residual compound or ethanol in the inoculum.

### Statistical analysis.

Data from all experiments are reported as means of at least three independent experiments, except where otherwise stated, and error bars represent the standard errors of the means (SEM). Data analysis was performed with the GraphPad Prism software 9.3.1 (GraphPad Software Inc., CA), and statistical differences were calculated using one-way or two-way analysis of variance (ANOVA) tests, followed by Bonferroni’s *post hoc* test. Samples were considered statistically different when *P* values were <0.05.
